# NMR spectroscopy of live human asthenozoospermic and normozoospermic sperm metabolism

**DOI:** 10.1530/RAF-21-0101

**Published:** 2022-03-21

**Authors:** Steven Reynolds, Sarah J Calvert, Stephen J Walters, Martyn N Paley, Allan A Pacey

**Affiliations:** 1 Imaging Sciences, Department of Immunity, Infection and Cardiovascular Disease, University of Sheffield, Royal Hallamshire Hospital, Sheffield, UK; 2 Department of Oncology and Metabolism, University of Sheffield, The Jessop Wing, Tree Root Walk, Sheffield, UK; 3 School of Health Related Research, University of Sheffield, Regent Court, Sheffield, UK

**Keywords:** metabolomics, human sperm, asthenozoospermia, motility, nuclear magnetic resonance spectroscopy, NMR

## Abstract

Sperm motility varies between ejaculates from different men and from individual men. We studied normozoospermic and asthenozoospermic ejaculates after density-gradient centrifugation washing (DCG, 80/40%) and compared high- (80%) and low (40%)-motility sperm populations within the same sample. Our objective was to identify differences in endogenous metabolomes and energy metabolism in relation to sperm motility. ^1^H-Nuclear Magnetic Resonance spectroscopy (NMR) measured the endogenous metabolome of live human sperm. Incubating sperm with ^13^C-labelled substrates detected energy metabolism by ^13^C-NMR. The study examined 850 ejaculates and diagnosed asthenozoospermia in 6.1%. DGC was used to wash 160 normozoospermic (N) and 52 asthenozoospermic (A) ejaculates to recover high-motility sperm from the pellet (80N/80A) and low motility from the interface (40N/40A). ^1^H-NMR spectra, 45(N) and 15(A), were binned and the integrals normalised by sperm concentration. Sperm from 126(N) and 36(A) ejaculates were incubated with either ^13^C-glucose, ^13^C-fructose or ^13^C-pyruvate. ^13^C-NMR lactate and bicarbonate integrals were normalised by motile or vital sperm concentrations. ^1^H-NMR spectra choline integrals from the 80A population were significantly lower than the 80N, *P*  < 0.0001. ^13^C-substrate conversion to lactate was significantly higher for 40A sperm than 80A sperm when normalised by motile sperm concentration. Bicarbonate integrals were sporadically observed. Sperm from asthenozoospermic ejaculates had similar glycolytic requirements to normozoospermic ones, with larger differences observed between 40 and 80% sperm populations. Higher lactate levels produced by 40% sperm may indicate that impaired sperm motility is due to dysregulated energy metabolism. The alteration in choline metabolism provides opportunities to understand the aetiology of asthenozoospermia.

**Lay summary:**

How well sperm swim (motility) varies between ejaculates from different men? Normal sperm motility is beneficial to conception and some men diagnosed with infertility have low sperm motility. Sperm metabolise molecules to produce the energy required for motility. We measured concentrations of molecules within sperm and metabolism of molecules given to sperm and related these to the proportion of motile sperm. The study examined 850 sperm samples and found low motility in 6.1%. Metabolism of molecules given to sperm was similar between low and normal motility sperm samples. However, when the most motile sperm were separated from the rest, they were more efficient in metabolising these molecules to achieve motility. Lower concentrations of a molecule called choline were found in low-motility sperm samples compared to normal samples. Choline is associated with cell membranes, energy metabolism and oxidative stress, which may give opportunities to understand the causes of low motility.

## Introduction

Asthenozoospermia is defined as <32% progressive sperm motility in semen, and while it can be readily identified at semen analysis, the underlying causes are not known (WHO 2010). A number of studies have examined asthenozoospermic sperm lysate samples for genomic (Heidary *et al.* 2020, Tu *et al.* 2020), proteomic (Cao *et al.* 2018) and metabolic defects (Paiva *et al.* 2015, Zhao *et al.* 2018). These have shown broad differences relating to energy production, stress response and tail structure pathways, but there is large variability between different studies.

Sperm metabolism is a dynamic process and a range of different metabolites are used by sperm to generate the ATP necessary to sustain motility. The roles of glycolysis (defined here as the conversion of glycolytic substrates, for example, glucose, fructose or pyruvate, to lactate) and oxidative phosphorylation (OxPhos) as ATP-generating pathways in sperm have been debated over many decades (Ruiz-Pesini *et al.* 2007, Piomboni *et al.* 2012, du Plessis *et al.* 2015). This is in part due to metabolic differences that can be observed between sperm of different species and the use of different substrates to probe these metabolic pathways. This is further confounded by conducting these experiments in various incubation media designed to mimic environments within the female reproductive tract. Despite this, many studies on human sperm show they favour glycolysis over OxPhos, although the role of the latter is acknowledged (Ruiz-Pesini *et al.* 2007, Piomboni *et al.* 2012, du Plessis *et al.* 2015). Examining the metabolism of live sperm may reveal temporal changes in sperm after ejaculation, and previous work by us showed sperm from normozoospermic samples metabolising glucose, fructose and pyruvate to lactate and bicarbonate (Calvert *et al.* 2019). Nuclear Magnetic Resonance spectroscopy (NMR) has the advantage that it can preserve sperm function during biochemical measurements made in real-time and under a variety of experimental conditions.

Density-gradient centrifugation (DGC) separates sperm from seminal plasma into high- and low-quality populations. Whilst differentiating in a number of physiological sperm traits, i.e. viability, morphology and DNA fragmentation, the method provides a means to creating sperm populations that differ in motility. In a previous study, we applied this method to normozoospermic ejaculates and measured the endogenous sperm metabolome of higher and lower motility populations by ^1^H-NMR (Reynolds *et al.* 2017*a*). This showed that the integrals from both the choline and lactate/lipid regions of the NMR spectrum were significantly higher in sperm with poorer motility compared to those with higher motility. Similarly, sperm metabolism of exogenous ^13^C-isotopically labelled molecules (e.g. glucose, galactose, pyruvate) was examined with ^13^C-NMR (Calvert *et al.* 2019) to estimate rates of metabolism. Contrary to expectation, the production of lactate (glycolysis) was significantly higher in sperm with poorer motility than the higher motility population by DGC. The generation of bicarbonate, an indicator of oxidative phosphorylation, was only observed occasionally.

Our previous studies were designed to identify metabolic differences between sperm of normozoospermic ejaculates that had been separated into high- and low-motility populations by DGC (Reynolds *et al.* 2017*a*, Calvert *et al.* 2019). Here, we extend these studies to also examine DGC-separated sperm from asthenozoospermic ejaculates. We hypothesise that ^1^H and ^13^C-NMR will identify significantly different endogenous and exogenous metabolomes between high- and low-motility sperm from asthenozoospermic ejaculates compared to normozoospermic ejaculates. Our first objective was to use ^1^H-NMR to compare the endogenous metabolomes of high- and low-quality sperm from asthenozoospermic and normozoospermic ejaculates. Secondly, we aimed to use ^13^C-NMR to measure how ^13^C-labelled metabolites are metabolised by high- and low-quality sperm from asthenozoospermic and normozoospermic ejaculates.

## Materials and methods

### Semen sample donation and analysis

Semen samples were obtained from men attending the Andrology Laboratory (Jessop Wing, Sheffield Teaching Hospitals NHS Foundation Trust, Sheffield, UK). All samples were produced on site and consent was given for the remaining ejaculate to be used in this study, after routine semen analysis. Ethical approval for this part of the study was given by the North of Scotland Research Ethics Committee (Reference: 16/NS/009) with clinical governance provided by Sheffield Teaching Hospitals NHS Foundation Trust (ref STH19095). All men recruited were asked about lifestyle factors that could influence semen quality (see Questionnaire template in Supplementary material, see section on supplementary materials given at the end of this article). A Fisher’s exact test was used for statistical testing of patient data.

To be included in the study, the experiment had to be started within 1 h of ejaculation, with a minimum of 25 million sperm present within the semen. Semen samples were classified as either asthenozoospermic (WHO 2010: progressive motility <32%) or normozoospermic (defined by the authors as progressive motility >40% to provide clear separation from the asthenozoospermia group).

### Sperm preparation

**Figure 1:**
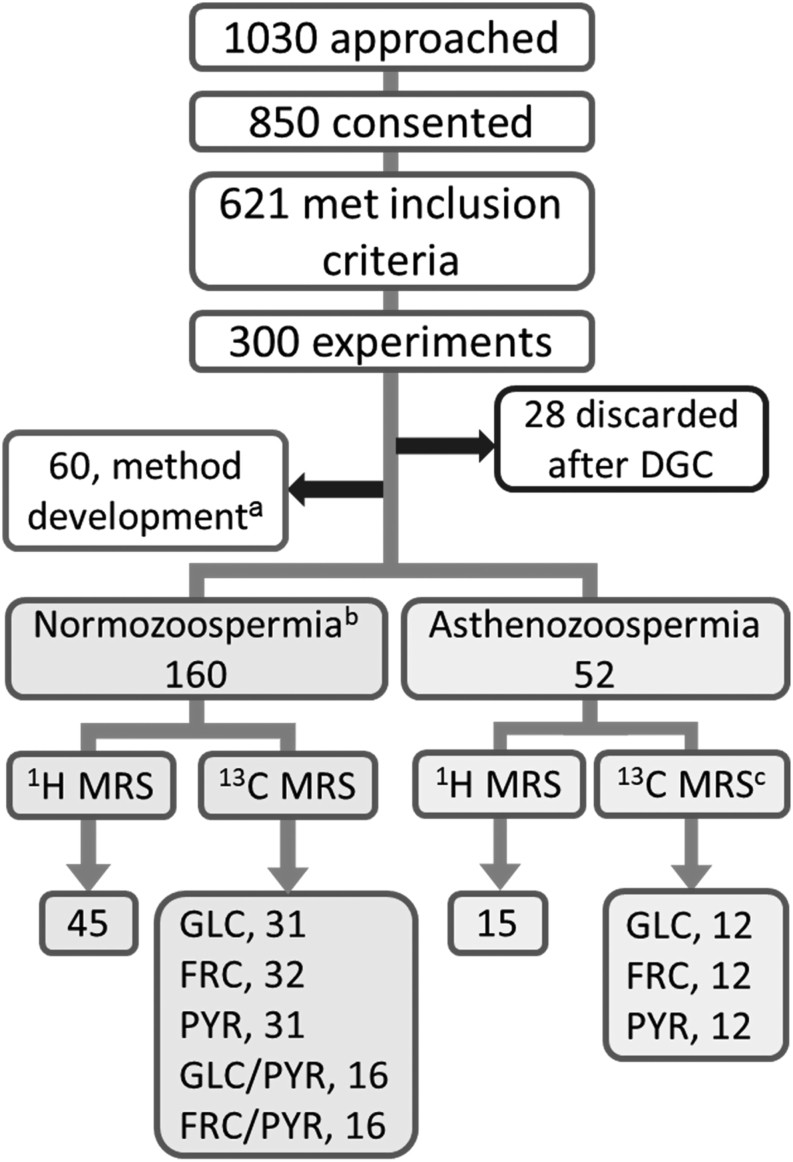
Number of men approached, consented and semen sample used for the study. GLC, ^13^C_u_-glucose; FRC, ^13^C_u_-fructose; PYR, ^13^C_1_-pyruvate. ^a^Ejaculates used for method development and washed with 40% Percoll/PBS only (Calvert *et al.* 2019); ^b^11 ejaculates were used for both ^1^H and ^13^C MRS experiments; ^c^1 sample randomly removed from analysis in order to balance groups.

Individual ejaculates were used for each experiment (i.e. no pooling). The protocols used to isolate sperm from seminal plasma have previously been published for ^1^H-NMR (Reynolds *et al.* 2017*a*) and ^13^C-NMR (Calvert *et al.* 2019). Briefly, approximately 1 mL of liquefied semen was layered on top of 40% (1.8 mL) and 80% (1.4 mL) Percoll/PBS solution (total DGC volume 3.2 mL, Percoll, GE Healthcare Life Sciences) in a 13 mL polypropylene tube with ventilation cap (Sarstedt, Leicester, UK). These tubes were then centrifuged for 20 min at 300 ***g*** to produce a population of sperm trapped at the 40/80% interface (termed 40% sperm population and typically with low motility) and those pelleted at the bottom of the tube (termed 80% sperm population and typically with high motility). These two populations of sperm were recovered and resuspended in PBS at least three times their recovered volume before being centrifuged again for 10 min at 500 ***g***. To further minimise seminal plasma contamination in the ^1^H-NMR spectrum, there was an additional centrifugation step with the supernatant removed and the sperm suspended in 600–1000 µL of fresh PBS followed by centrifugation at 500 ***g*** for 20 min (Reynolds *et al.* 2017*a*). The resulting pellets of both sperm populations were resuspended in PBS to yield a final volume of 600 µL before either ^1^H-NMR analysis or incubation with ^13^C-substrates incubation. The target concentration for experiments with either normozoospermic or asthenozoospermic ejaculates was ^1^H-NMR, ~40 × 10^6^/mL (min 16 × 10^6^/mL); ^13^C-NMR, ~30 ×10^6^/mL (min 11 × 10^6^/mL). Where possible, higher sperm concentration samples (>50 × 10^6^/mL) were diluted and sub-divided to perform separate parallel experiments. Conversely, when the 40%/80% sperm populations were below the target concentration, then the samples were resuspended in the minimum required volume (^1^H, 450 mL; ^13^C, 600 mL) to maximise the concentration while allowing the experiment to proceed. Eleven ejaculates were used for both ^1^H-NMR and incubation with ^13^C-substrates (Fig. 1). Sperm used in the analysis of ^13^C-NMR incubation samples were all from different men (i.e. no two substrates were compared for sperm from the same man). Thus, during these experiments, four different populations of sperm were obtained and examined by ^1^H- and ^13^C-NMR: those (i) from the 40/80% interface (40N); or (ii) from the pellet at the bottom of the centrifuge tube (80N) of normozoospermic ejaculates; and (iii) from the 40/80% interface (40A); or (iv) from the pellet at the bottom of the centrifuge tube of asthenozoospermic ejaculates (80A). Samples used in ^1^H-NMR experiments were scanned immediately, whereas those incubated with ^13^C substrates were prepared as described below.

### Baseline sperm/semen measurements

After diagnostic semen analysis had been completed, further measurements of sperm concentration and motility were obtained by Computer-Aided Sperm Analysis (SCA, Microptic SL, Barcelona, Spain, see Reynolds *et al.* (2017*a*) for microscope details) at three time points, as previously described (Calvert *et al.* 2019): (i) in semen within 1 h of ejaculation; (ii) following DGC; and (iii) after the experiment had been completed (~3 h for the ^1^H NMR cohort and ~4 h for the ^13^C-NMR cohort). Vitality measurements were also performed at time points (ii) and (iii) (Calvert *et al.* 2019). Measurements for semen volume, pH and sperm morphology were obtained from the initial semen analysis report and were not repeated. The concentration of non-sperm cells in the washed sperm populations was determined as previously described (Calvert *et al.* 2019). Baseline semen/sperm data were compared using either a Student’s unpaired *t*-test or Wilcoxon signed-rank test based on the outcome of D’Agostino and Pearson normality of distribution tests using Matlab (R2018a). All results are quoted as mean ± s.e.m. unless otherwise stated.

### NMR experiments and data analysis

A 9.4T (400MHz) Bruker Avance III NMR spectrometer (Bruker BioSpin GmbH, Karlsruhe, Germany), with a 5 mm broadband observe probe was used for all experiments.

### ^1^H-NMR cohort experiments

Two NMR tubes were prepared containing a 350 µL aliquot of washed ‘40%’ or ‘80%’ sperm population that was added to a 5 mm H_2_O magnetic susceptibility matched NMR tube (Shigemi, BMS005J, Tokyo, Japan) along with 20 µL of D_2_O (Sigma Aldrich). NMR acquisition, processing and analysis have been detailed in Reynolds *et al*. (2017*a*). From each ejaculate, both sample tubes were scanned sequentially in random order at 37°C using an excitation sculpting water suppression sequence, with the other NMR tube (40% or 80%) kept at room temperature until it could be scanned. Peak alignment was achieved by spectral referencing (choline;^ 1^H, δ = 3.23 ppm) and visual inspection of the spectra.

Spectra were binned at 0.04 ppm, with the water peak between 4.5 and 5.2 ppm removed (Matlab R2018b, Mathworks, Natick, MA, USA). The binned spectral integrals were then normalised to total sperm concentration and compared using a two-way ANOVA with a Bonferroni multiple comparison test, where *P*  < 0.05 was regarded as significant. All results are quoted as mean ± s.e.m. unless otherwise stated.

### ^13^C-NMR cohort experiments

Sperm metabolism was examined by individually incubating 500 µL of 40 and 80% sperm populations from the washed sample with 15 µL of penicillin/streptomycin antibiotics and 40 µL of 100 mM ^13^C labelled substrate (^13^C_u_-glucose, ^13^C_u_-fructose or ^13^C_1_-pyruvate, Sigma Aldrich). Where two ^13^C-labelled substrates were used (i.e. ^13^C_u_-glucose\^13^C_1_-pyruvate and ^13^C_u_-fructose\^13^C_1_-pyruvate), 20 µL of the above stock solutions were added instead. All samples were incubated for 4 h at 37°C and then frozen at −80°C until NMR analysis. Frozen samples were thawed and 380 µL was placed in a 5 mm NMR tube (Norell, Morganton, NC, USA) with 20 µL D_2_O (Sigma Aldrich) and 10 µL of 200 mM ^13^C-urea (chemical shift and concentration reference, Sigma Aldrich) for NMR analysis.

Details of ^13^C-NMR acquisition, processing and analysis have previously been published (Calvert *et al.* 2019). Spectra were acquired using a ^13^C{^1^H} inverse-gated pulse sequence and referenced to the urea signal (δ = 165.5 ppm). ^13^C-spectra peaks were integrated with predefined chemical shift integral ranges for (^13^C_1_-lactate ^13^C_1_-pyruvate, ^13^C-urea, ^13^C-bicarbonate, ^13^CO_2_, ^13^C_u_-glucose, ^13^C_u_-fructose, ^13^C_2_-lactate, ^13^C_3_-lactate). The integrals for bicarbonate and carbon dioxide were summed to account for the biological equilibrium in which these molecules exist.

The amount of ^13^C_1_-labelled lactate derived from each of the substrate(s) incubations was correlated to vital or motile sperm concentration and non-sperm concentration using a linear model. Outliers to the linear regression, defined as >1.5× interquartile range were removed from both axes to prevent extreme values overly influencing the fit. The value of r^2^ and significance of the correlation (corrected for multiple comparisons) are reported for the fit. A Pearson’s regression fit was also performed for total sperm concentration vs non-sperm cell concentration. Statistical differences between the four different sperm populations (40N, 80N, 40A, 80A) were tested in Matlab using a Kruskal–Wallis non-parametric test and a multi-comparison Bonferroni* post hoc* test (abbreviated to KW-B), with *P* ≤ 0.05 used as the significance threshold. All results are quoted as mean ± s.e.m. unless otherwise stated.

## Results

A total of 1030 men were approached to participate in this study of which 850 consented to their semen to be used. Of these, 621 samples met the inclusion criteria with 569 classified as normozoospermic. The remaining 52 ejaculates were classified as asthenozoospermic giving a prevalence of asthenozoospermia of 6.1% (52/850) of ejaculates. The asthenozoospermic ejaculate cohort included 10 diagnosed as oligoasthenozoospermia by concentration (WHO 2010) but containing over 25 × 10^6^ sperm after analysis. For each NMR cohort, these were: ^1^H, *n*  = 1; ^13^C-Glc, *n*  = 3; ^13^C-Frc, *n*  = 4; ^13^C-Pyr, *n*  = 2. A total of 60 ejaculates were used for ^1^H-NMR analysis and 162 ejaculates for ^13^C-NMR experiments (see Fig. 1).

There were no significant differences between the asthenozoospermic and normozoospermic groups of men in any of the lifestyle factors measured (e.g. age, having previously conceived. See Supplementary Tables 1, 2, 3, 4, 5 and 6 for full analysis list at https://figshare.com/s/740424eb8c7612b17e5f), except in the ^13^C-NMR cohort where there was a small but significant reduction in BMI for men who provided asthenozoospermic ejaculates compared to those who produced normozoospermic ones (Supplementary Table 1: median (range): 25.0 (17.7–30.9) vs 25.8 (17.6–41.8), *P*  = 0.025, Wilcoxon rank-sum test). In addition, while the ethnicity of men who produced normozoospermic ejaculates was in line with national averages, a higher proportion of men of Asian, Black, mixed or other ethnicities produced asthenozoospermic ejaculates (see Supplementary Table 7) in our sample.

### Semen analysis and sperm quality measures

As expected by the study design, there was a significant difference in progressive motility for normozoospermia (>40% motility) and asthenozoospermia ejaculates (see Table 1). Comparing the cohorts, there was no significant difference between the time of semen production and completion of semen analysis that could have contributed to this (see Table 1). The concentration of sperm in semen was significantly lower for asthenozoospermia than normozoospermia ejaculates (see Table 1); therefore, ^1^H and ^13^C integrals used in the analysis were normalised by sperm concentration (see below for specific concentration definitions). Only the ^13^C cohort, and not the ^1^H cohort, showed significantly lower normal sperm morphology for the asthenozoospermic populations, see Table 1 (Supplementary Tables 8 and 9 for a per substrate breakdown).

**Table 1:** Semen baseline parameters measured by the Andrology unit at the Jessop Wing. Samples were separated into two cohorts for ^1^H and ^13^C-NMR.

Parameter	Normozoospermia	Asthenozoospermia	*P*-value
^1^H-NMR cohort			
*n*	45	15	
Abstinence^a^, days	5.4 ± 0.3	5.7 ± 1.2	0.47
Analysis delay^b^, min	24.7 ± 1.4	27.1 ± 2.9	0.42
Volume, mL	4.4 ± 0.3	5.0 ± 0.5	0.29
pH (semen)	8.11 ± 0.04	8.07 ± 0.05	0.49
Concentration, 10^6^/mL	96.9 ± 8.0	41.9 ± 6.4	< 0.0001^wil^
Morphology, %	7.9 ± 0.8	7.2 ± 1.4	0.66
Motility, % (range)			
TM	62.1 ± 1.1	31.4 ± 2.0	< 0.0001^wil^
PR	59.5 ± 1.3	24.2 ± 1.5	< 0.0001^stt^
Viscosity^c^	82% (37) normal	80% (12) normal	1.0
Agglutination^d^	0%	20% (3 cases)	0.003^wil^
^13^C-NMR cohort			
*n*	126	36	
Abstinence^a^, days	4.8 ± 0.2	6.6 ± 0.9	0.23
Analysis delay^b^, min	24.9 ± 0.8	26.9 ± 1.8	0.43
Volume, mL	4.3 ± 0.1	4.3 ± 0.3	0.95
pH (semen)	8.08 ± 0.02	8.15 ± 0.04	0.17
Concentration, 10^6^/mL	84.3 ± 5.4	44.1 ± 6.4	< 0.0001^wil^
Morphology, %	7.4 ± 0.6	5.8 ± 1.1	0.03^ wil^
Motility, % (range)			
TM	62.7 ± 0.9	30.2 ± 1.1	< 0.0001^wil^
PR	60.0 ±0.9	24.7 ± 1.2	< 0.0001^wil^
Viscosity^c^	78% (98) normal	67% (24) normal	0.19
Agglutination^d^	4% (5)	6% (2)	0.66

^a^Abstinence – where >2 days was reported this was set to 2 days; ^b^Analysis delay represents the time elapsed between semen ejaculation and commencing Andrology analysis; ^c^Viscosity was graded as either normal or high; ^d^Agglutination presented as number of cases where some degree of agglutination was noted (isolated, moderate, widespread). Numbers are presented as either mean ± s.e.m.or percentage number of cases (absolute number in parenthesis). Statistical tests (selected on the outcome of a D’Agostino–Pearson normality test): ^stt^, unpaired Student’s *t*-test; ^wil^, Wilcoxon rank sum; ^Fish^, Fisher test.

**Figure 2:**
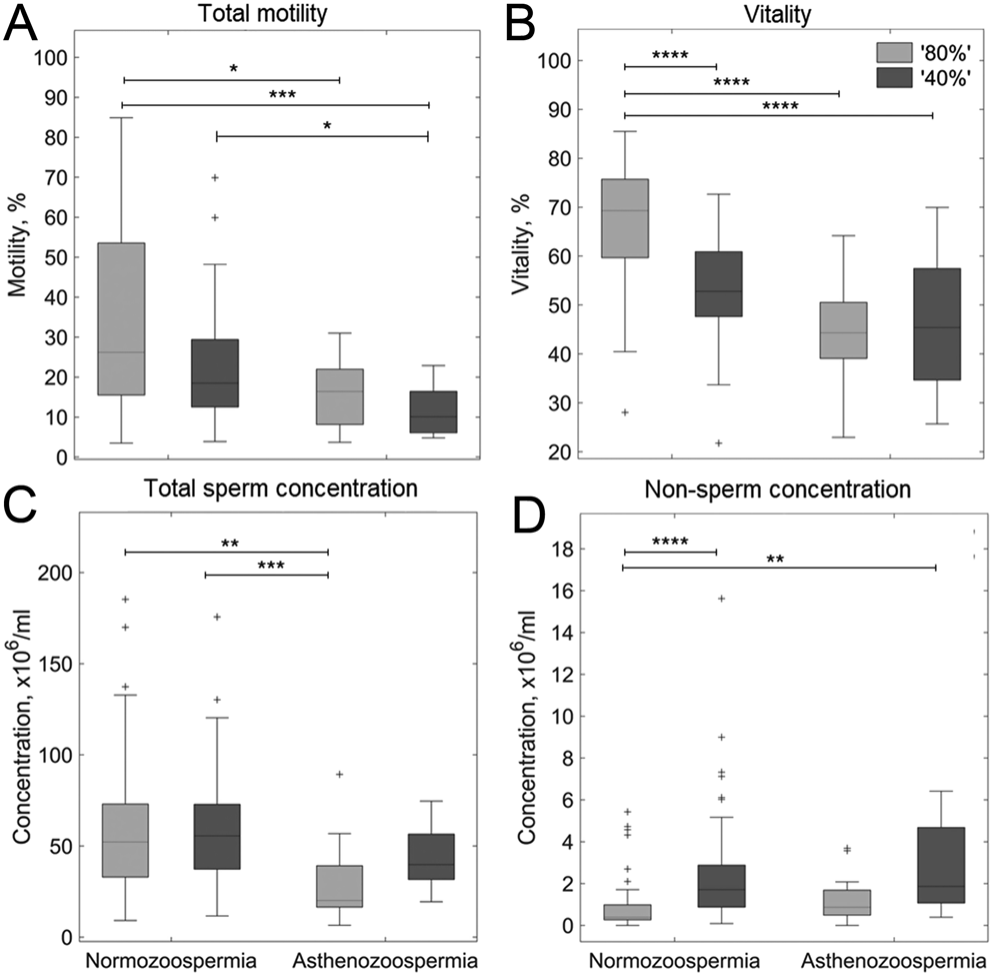
^1^H-MRS cohort (measured at time zero) sperm physiology measure after DGC separation into ‘40% (dark grey)’ and ‘80% (light grey)’ population derived from men diagnosed with normozoospermia or asthenozoospermia, see Fig. 1 for number of samples; (A) total motility (PureSperm wash), (B) vitality (PureSperm wash), (C) concentration (PBS), (D) concentration of non-sperm cells. Kruskal–Wallis with Bonferroni p*ost hoc* test performed on all datasets. ^*^P  < 0.05; ^**^P  < 0.01; ^***^P  < 0.001; ^****^P  < 0.0001.

**Figure 3:**
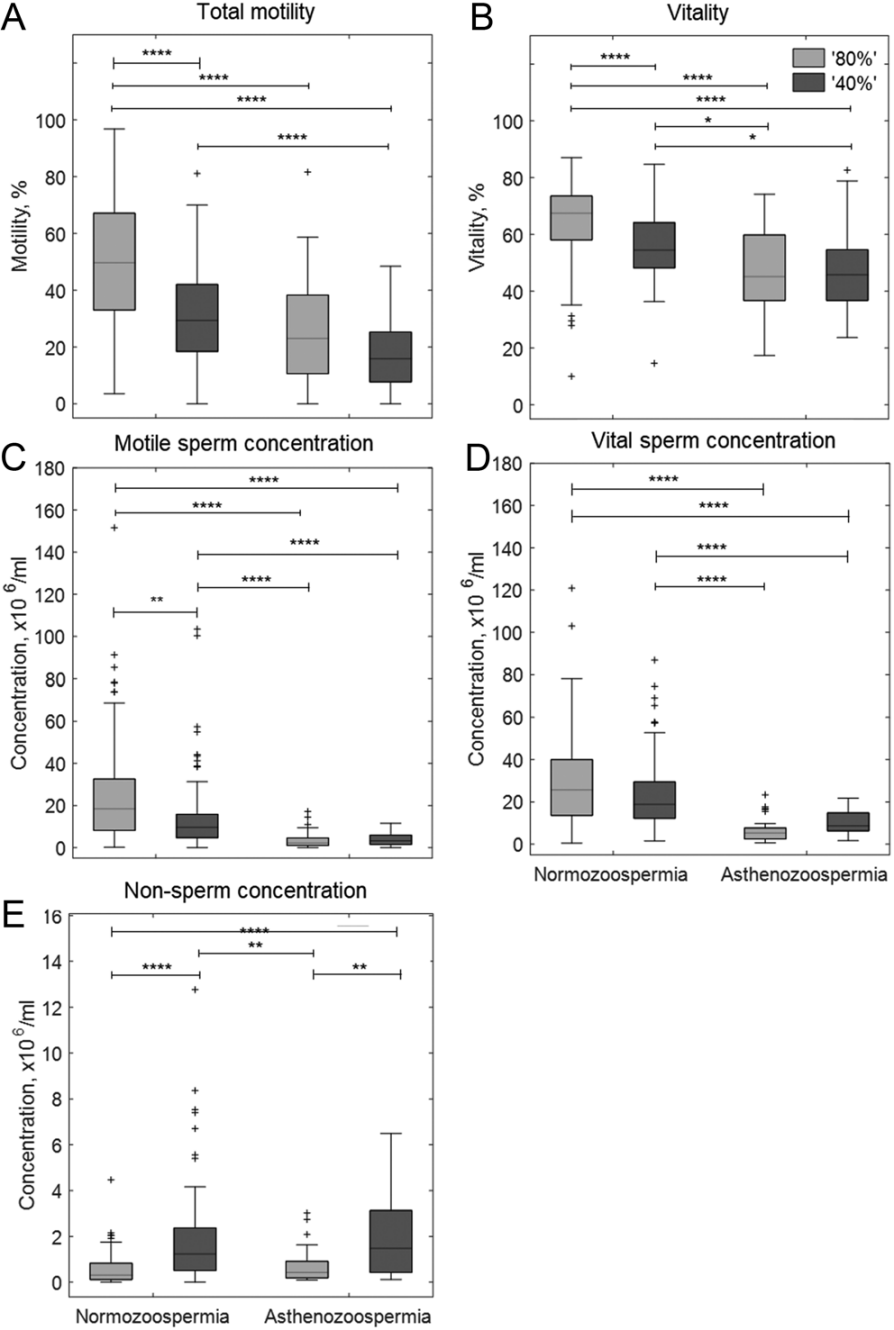
^13^C MRS cohort (measured at time zero) sperm physiology measured after DGC separation into ‘40% (dark grey)’ and ‘80%’ (light grey) population derived from men diagnosed with normozoospermia or asthenozoospermia, see Fig. 1 for number of samples; (A) total motility (PureSperm wash), (B) vitality (PureSperm wash), (C) motile sperm concentration, (D) vital sperm concentration, (E) concentration of nonsperm cells. Kruskal–Wallis with Bonferroni *post hoc* test performed on all datasets. ^*^P  < 0.05; ^**^P  < 0.01; ^***^P  < 0.001; ^****^P  < 0.0001.

The 80N population had the highest proportion of motile sperm and there was a decreasing trend in percent motility 80N > 40N ≈ 80A > 40A in ejaculates used for both ^1^H (Fig. 2) and ^13^C analysis (Fig. 3). Similarly, sperm vitality was highest for 80N followed by 40N sperm populations and lowest in both the asthenozoospermic populations, although the vitality of 40A and 80A sperm populations was not significantly different from each other. In the ^1^H cohort, total sperm concentrations from the normozoospermic populations were significantly higher than those for 80A populations (see Fig. 2). For the ^13^C cohort, normozoospermic population concentrations were significantly higher than those for asthenozoospermic populations (80N, 43.1 ± 3.8 × 10^6^/mL; 40N, 42.4 ± 3.8 × 10^6^/mL; 80A, 13.5 ± 2.3 × 10^6^/mL; 40A, 23.1 ± 3.8 × 10^6^/mL, mean ± s.e.m). Calculation of the motile and vital sperm concentrations for the ^13^C cohort showed further significant differences (Fig. 3).

After ^1^H-NMR acquisition or ^13^C-substrate incubation of both ‘40%’ and ‘80%’ sperm populations, the sperm concentration, motility and vitality were recorded. The significant differences observed for these parameters largely replicated those observed at time 0 (see Supplementary Figs 1 and 2). While generally low (<2 × 10^6^/mL), the median non-sperm cell (leukocytes + epithelial cells) concentration was higher in ‘40%’ sperm populations compared to ‘80%’ sperm populations, see Fig. 2 (^1^H-cohort), Fig. 3 (^13^C-cohort), as well as Supplementary Fig. 3 for per substrate breakdown.

### NMR analysis

#### ^1^H-NMR cohort experiments

**Figure 4:**
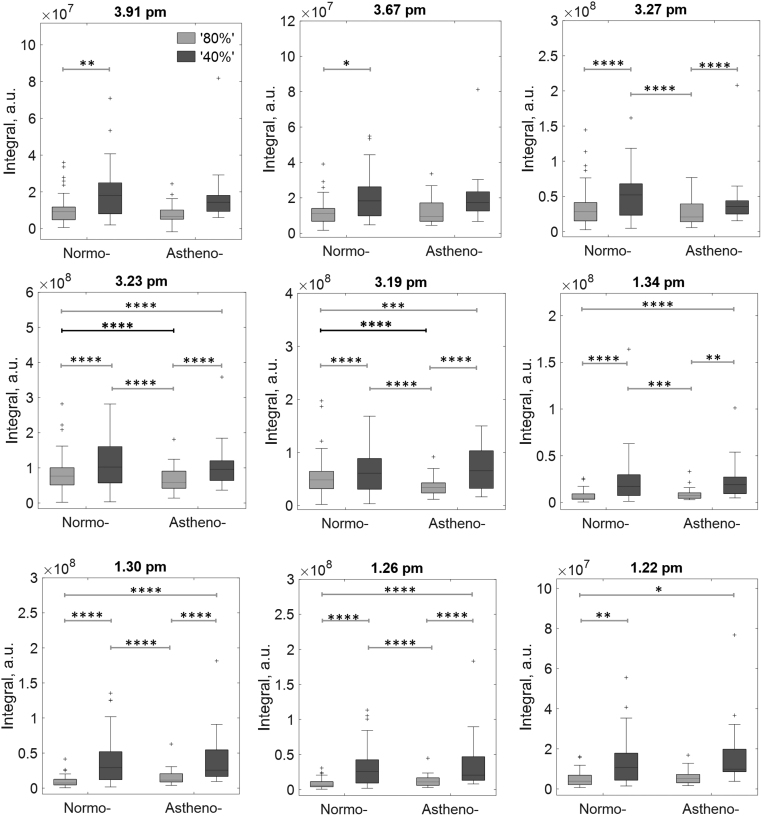
Boxplot of integrals at different bin chemical shift values of the ^1^H-MRS spectrum (each integral normalized to sperm concentration). A two-way ANOVA with Bonferroni *post hoc* analysis was applied to identify significant differences between the four groups (80N, 40N, 80A, 40A. See Figure 1 for number of samples). Only those groups where the bin integrals (indicated at the top of each plot) were significantly different are shown. Bars at the top of the figure show comparisons between groups: ^*^P  < 0.05; ^**^P  < 0.01; ^***^P  < 0.001; ^****^P  < 0.0001 (see Table TX for exact *P* -values). Bin integrals located at 3.23 and 3.19 ppm also show in bold black are significant differences between 80N and 80A sperm samples.

Significant differences were found between ‘40%’ and ‘80%’ sperm populations in two main regions of contiguous bins: (i) 3.19–3.26 ppm, a chemical shift associated with choline derivatives and (ii) 1.22–1.34 ppm, a chemical shift associated with lactate as well as the aliphatic chain of lipids. Significant differences were more often found between ‘40%’ and ‘80%’ sperm populations than between asthenozoospermic (*n*  = 15) and normozoospermic (*n*  = 45) samples (Fig. 4). Within normozoospermic and asthenozoospermic populations, all bins located at 3.91, 3.67, 3.27, 3.23, 3.19, 1.34, 1.30 and 1.26 ppm showed significant differences between 40N and 80N populations and between 40A and 80A populations (except for bins at 3.91 and 3.67 ppm).

The only significant differences between the same populations (80A and 80N) of asthenozoospermic and normozoospermic samples were between bins 3.19 and 3.23 ppm (shown by bold significance bars, see Fig. 4). Additionally, 80A populations were significantly lower than 40N populations at locations 3.27, 3.23, 3.19, 1.34, 1.30 and 1.26 ppm. Finally, 80N populations were significantly lower than 40A populations at locations 3.23, 3.19, 1.34, 1.30, 1.26 and 1.22 ppm. See Supplementary Table 10 for exact *P* -values.

#### ^13^C-NMR cohort experiments

##### Normalised by vital sperm concentration

**Figure 5:**
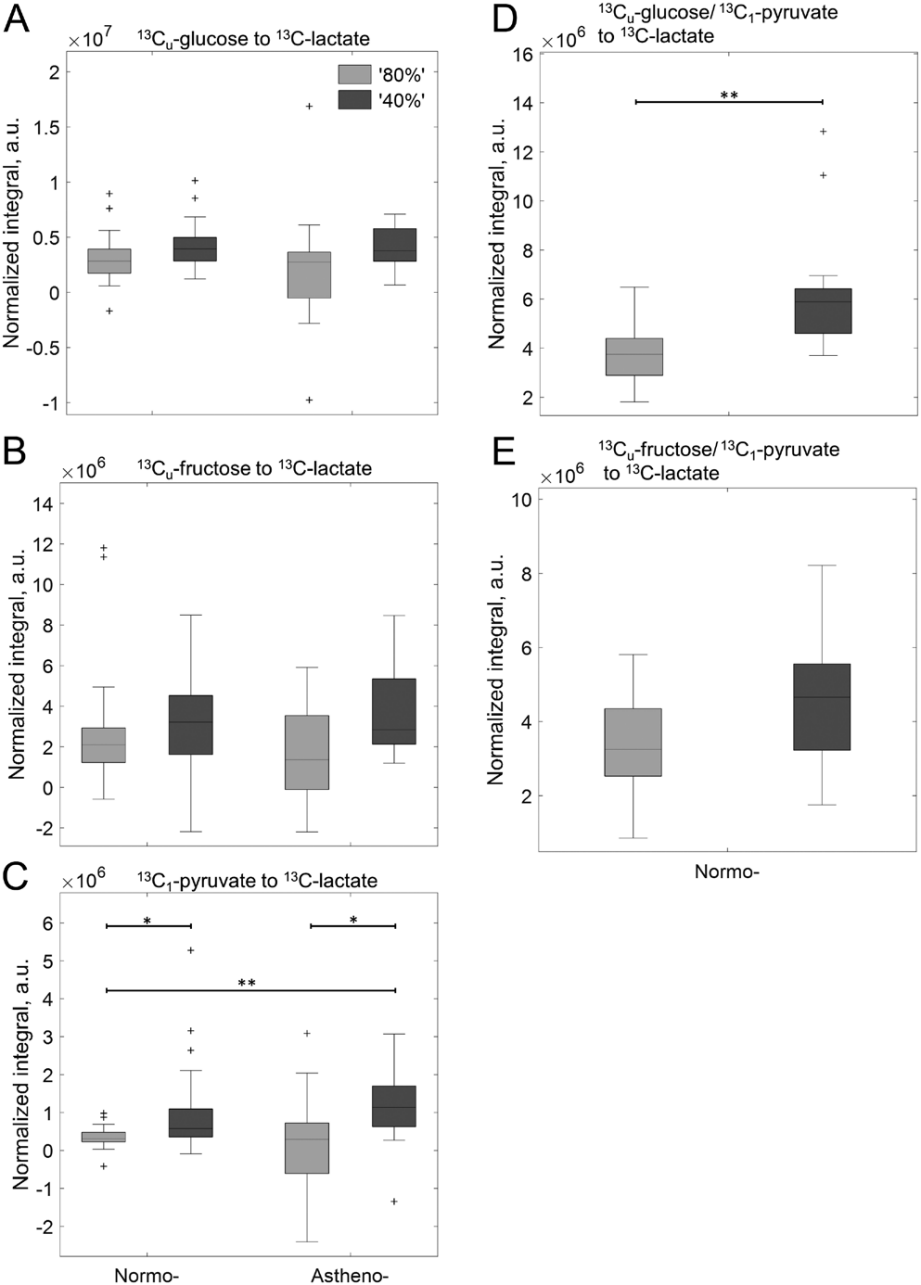
^13^C_1_-lactate integrals normalized by vital sperm concentration for DGC washed sperm populations (‘40%’ – dark grey, ‘80%’ – light grey) derived from men diagnosed with normozoospermia or asthenozoospermia. See Figure 1 for number of samples. (A) ^13^C_u_-glucose, (B) ^13^C_u_-fructose, (C) ^13^C_1_-pyruvate, (D) ^13^C_u_-glucose/^13^C_1_-pyruvate, (E) ^13^C_u_-fructose/^13^C_1_-pyruvate. Significant difference identified from a Kruskal–Wallis with Bonferroni *post hoc* test. ^*^P  < 0.05; ^**^P  < 0.01.

There were no significant differences in vitality normalised ^13^C_1_-lactate integrals for ^13^C_u_-glucose and ^13^C_u_-fructose or ^13^C_u_-frutose/^13^C_1_-pyruvate incubations between any of the sperm populations (Fig. 5A, B and E). In contrast, 80N populations incubated with ^13^C_1_-pyruvate or ^13^C_u_-glucose/^13^C_1_-pyruvate combinations produced significantly lower ^13^C_1_-lactate integrals than 40N populations (*P*  = 0.018 (KW-B) and *P*  = 0.0011 (Mann–Whitney *U*-test)), Fig. 5C and D). Similarly, 80A populations incubated with ^13^C_1_-pyruvate produced significantly lower ^13^C_1_-lactate integrals than 40A (*P*  = 0.046, Fig. 5C). Finally, 80N populations incubated with ^13^C_1_-pyruvate produced significantly lower ^13^C_1_-lactate integrals than 40A populations (*P*  = 0.006, Fig. 5C). Glycolysis of ^13^C_u_-glucose or ^13^C_u_-fructose, as part of the combined incubation, leads to detectable quantities of ^13^C_1_-pyruvate. However, there were no significant difference in the amount of ^13^C_u_-pyruvate present in ‘40%’ or ‘80%’ populations.

##### Normalised by motile sperm concentration

**Figure 6:**
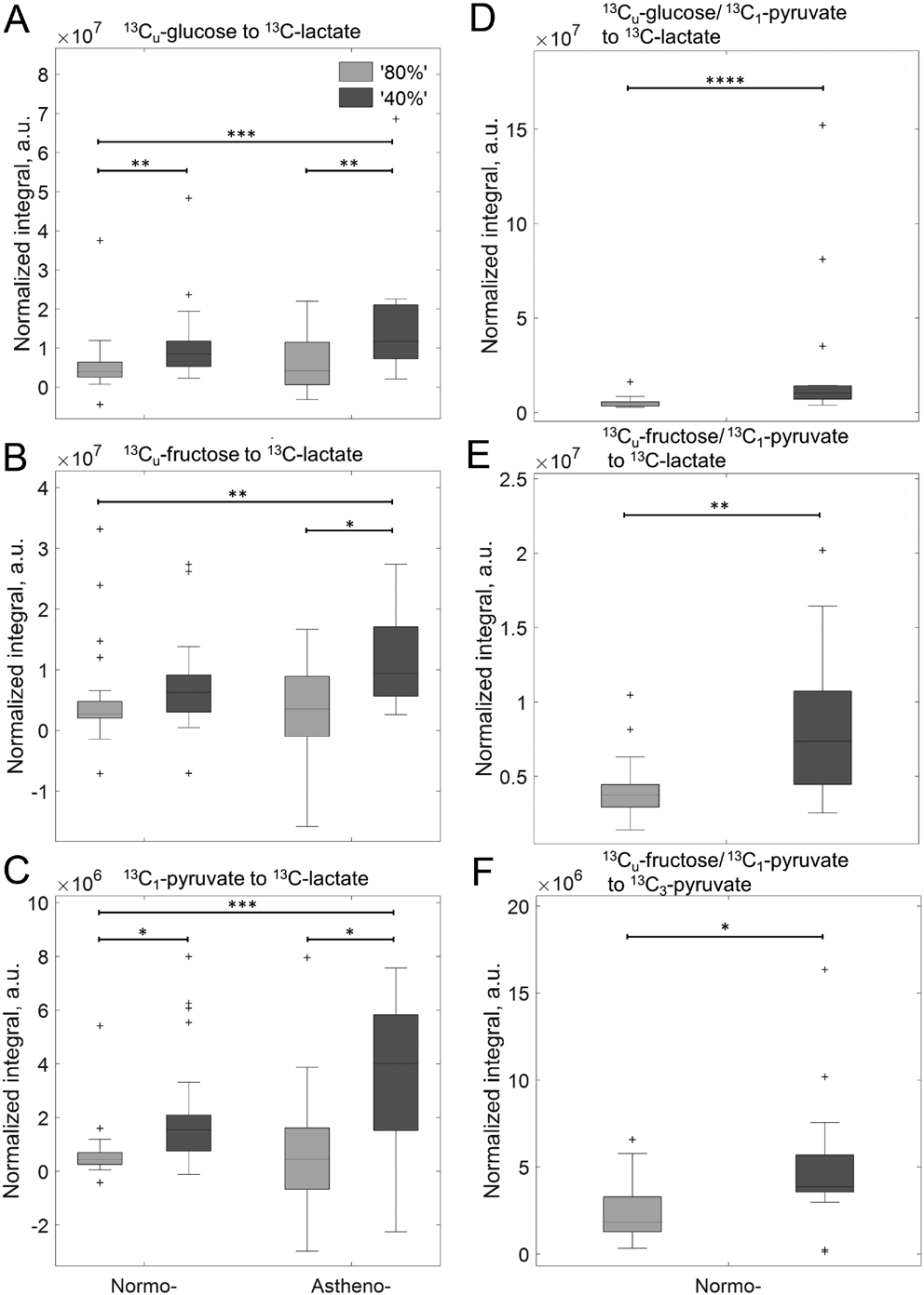
^13^C_1_-lactate (A, B, C, D and E) integrals normalized by motile sperm concentration washed sperm populations (‘40%’ – dark grey, ‘80%’ – light grey) derived from men diagnosed with normozoospermia or asthenozoospermia, see Figure 1 for number of samples. ^13^C_1_-lactate integral was measured from incubation performed with (A) ^13^C_u_-glucose, (B) ^13^C_u_-fructose, (C) ^13^C_1_-pyruvate or in combination, (D) ^13^C_u_-glucose/^13^C_1_-pyruvate and (E) ^13^C_u_-fructose/^13^C_1_-pyruvate. Conversion of ^13^C_u_-fructose to ^13^C_3_-pyruvate from ^13^C_u_-fructose/^13^C_1_-pyruvate incubation (F). Significant difference identified from a Kruskal–Wallis with Bonferroni *post hoc* test. ^*^P  < 0.05; ^**^P  < 0.01; ^***^P  < 0.001; ^****^P  < 0.0001.

Again, no significant differences were seen when comparing lactate integrals produced by either sperm populations between asthenozoospermic and normozoospermic samples. Normalising ^13^C_1_-lactate integrals by motile sperm concentration showed further significant differences between ‘80%’ vs ‘40%’ populations. In the case of ^13^C_u_-glucose incubations, ^13^C_1_-lactate was significantly lower for ‘80%’ populations compared to their corresponding ‘40%’ normo/asthenozoospermic populations (80N vs 40N, *P*  = 9.8 × 10^−4^; 80A vs 40A, *P*  = 0.045) (Fig. 6A). The 80N populations also had significantly lower ^13^C_1_-lactate than 40A (*P*  = 9.1 × 10^−4^). For ^13^C_u_-fructose incubations (Fig. 6B), the 40A population had significantly higher lactate than both the 80N and 80A (40A vs 80N, *P*  = 0.002 and vs 80A, *P*  = 0.029). When ^13^C_1_-lactate integrals derived from ^13^C_1_-pyruvate were normalised to motility, they were significantly lower for ‘80%’ populations compared to their corresponding ‘40%’ normo/asthenozoospermic populations (80N vs 40N, *P*  = 0.0016; 80A vs 40A, *P*  = 0.013) (Fig. 6C). This was comparable to vitality normalised data. Also, the 80N populations had significantly lower ^13^C_1_-lactate than 40A (*P*  = 0.0002). The combined incubations of ^13^C_u_-glucose/^13^C_1_-pyruvate and ^13^C_u_-frutose/^13^C_1_-pyruvate both showed significant differences for ^13^C_1_-lactate between the 80N and 40N populations (*P*  = 0.0003 and *P*  = 0.0028 (both Mann–Whitney *U*-test), Fig. 6D and E). The amount of pyruvate being formed from ^13^C_u_-fructose, as seen by the appearance of a ^13^C_3_-pyruvate peak, was significantly higher for 40N than 80N populations (*P*  = 0.022, Fig. 6F).

##### Substrate metabolism to bicarbonate

Bicarbonate was only observed sporadically irrespective of the incubation substrate, DGC separated populations or motility assessment. There were no significant differences in integrals found for the formation of bicarbonate/CO_2_ from any of the ^13^C-substrate(s) incubations (data not shown).

##### Sperm contribution examined by linear regression model

The lactate signal observed in the NMR spectrum was dependent on the concentration of sperm present (Calvert *et al.* 2019). Some non-sperm cells that passed through the washing process could potentially contribute to the metabolism observed. This possibility was examined for lactate using a linear regression model, see Supplementary material. For ^13^C_u_-glucose and ^13^C_u_-fructose-containing incubations, the model showed that in most cases, sperm concentration was the main factor responsible for the lactate signal (see Supplementary Table LMR1). ^13^C_1_-pyruvate showed an inconsistent fit or no fit between the lactate integral and sperm/non-sperm concentration, as previously shown (Calvert *et al.* 2019).

## Discussion

This is the first study to compare the metabolomes of live human sperm from asthenozoospermic and normozoospermic samples by ^1^H and ^13^C-NMR. Examining live sperm allows the observation of ongoing changes in metabolomes and the capability to use the sperm after analysis to monitor temporal changes in sperm metabolomes as a result of, for example, capacitation. Furthermore, metabolomics could be used in clinical analysis, where once a metabolomic assessment had been made, the same sperm could be used in assisted conception. Sperm motility varies considerably between ejaculates from different men and from the same man. Both ‘40%’ and ‘80%’ sperm populations were used from each sample to give a greater insight into the metabolic causes of poor motility sperm derived from the same ejaculate (40% vs 80% populations) in addition to sperm from ejaculates diagnosed with asthenozoospermia. The study approached over 1000 men and a high acceptance rate (82.5%) allowed us to collect as many potentially asthenozoospermic sample as possible. Of the 850 who consented for the study, 52 provided asthenozoospermic ejaculates giving an incidence of ~6%. This is similar to a recent re-evaluation based on WHO (2010) guidelines by Larach *et al.* (2013) which found 5.8% of samples to contain asthenozoospermia, oligoasthenozoospermia and oligoasthenoteratozoospermia.

Lifestyle and physical factors showed a small reduction in BMI for men who produced asthenozoospermic ejaculates in the ^13^C cohort. Previous large studies have been conducted into the effect of lifestyle factors on sperm motility, but these did not identify obesity or underweight as a risk factor (Povey *et al.* 2012, Jurewicz *et al.* 2014). Considering our sample size, coupled with the fact that BMI was only a significant factor (*P*  = 0.025) in the ^13^C group (and only for ^13^C_1_-pyruvate when stratified by substrate), it is unlikely that BMI plays a significant role. Compared to a national database of ethnicity (2011 UK census data, Supplementary Table 7), significantly more men who identified as black or minority ethic (BME) provided asthenozoospermic ejaculates in both the ^1^H and ^13^C cohorts. While there is evidence for global variation in male fertility, this is confounded by social and cultural barriers (Agarwal *et al.* 2015). Ours was a UK-based population and similar UK-based study found members of the BME community to be more likely to have a low motile sperm concentration (Povey *et al.* 2012). Whether ethnicity influences the metabolomics of sperm, possibly through the genomic differences, remains unanswered.

Overall, metabolite differences between ‘40%’ and ‘80%’ sperm populations were larger than the corresponding differences between sperm from the same populations of asthenozoospermic and normozoospermic ejaculates. However, some significant differences were found in ^1^H-NMR spectra between 80A and 80N populations at the 3.19 and 3.23 ppm integral bins. These regions contain free choline (3.21 ppm) and its derivatives. We found no significant differences for 40A and 40N, which suggests that poor-quality sperm metabolomes are indistinguishable regardless of whether they originate from asthenozoospermic or normozoospermic ejaculates. Many significant differences were found between 80N vs 40A and 80A vs 40N, but we think these differences largely reflect the differences between ‘40%’ and ‘80%’ sperm populations rather than being specific to asthenozoospermia.

The integrals in the 3.19 and 3.23 ppm bins were lowest for the 80A sperm. ^1^H-spectra showed several peaks in this region, generally the largest being at ~3.2 ppm with two peaks diminishing in size to the left, related to choline metabolism. However, other metabolites have been identified within this range (Paiva *et al.* 2015, Murgia *et al.* 2020), but the choline molecular group is likely the most prominent representing nine equivalent protons. Also, using NMR, compounds that are constrained within the cellular membrane (e.g. phosphatidylcholine) have broad peaks and, hence, reduced intensity compared to solubilised small molecules (Miller *et al.* 1996, Griffin *et al.* 2001, Ross & Bluml 2001). The observed choline signal is likely to be derived from cytosolic choline. Further work using multidimensional NMR could separate overlapping peaks and confirm the molecular assignment. Assuming choline is primarily responsible for the observed differences, our finding correlates with other studies that found decreased choline from seminal fluid samples from oligoasthenoteratozoospermia and oligoasthenozoospermia compared to normozoospermia (Boguenet *et al.* 2020, Mumcu *et al.* 2020).

There are many ways in which choline and its metabolites could influence sperm motility, as it is important for synthesising phosphatidylcholines for cell membranes, regulating lipid metabolism, protecting against oxidative stress and is a marker of cell proliferation (Infante 1987, Mumcu *et al.* 2020). For *in vivo* NMR studies, the magnitude of the choline peak is assumed to be a marker of cell membrane synthesis (Verma *et al.* 2016) and turnover. Low choline could directly affect membrane composition and reduce sperm motility. However, the phospholipid composition of normozoospermic and asthenozoospermic sperm was examined by Tavilani *et al.* (2007) who found that the content of phosphatidylcholine (PC) and phosphatidylethanolamine (PE) was not significantly lower for sperm from asthenozoospermic ejaculates.

A choline-deficient diet in rats leads to oxidative stress and tissue damage in kidney and heart (Repetto *et al.* 2010). If low choline observed in asthenozoospermic sperm increases oxidative stress, then the resultant lipid peroxidation could directly reduce sperm motility (Rao *et al.* 1989). Indeed, Tavilani *et al.* (2007) showed that a lipid peroxidation marker was significantly higher in sperm from asthenozoospermic ejaculates compared to normozoospermic ones. Many other studies have linked increased oxidative stress to asthenozoospermia (Khosrowbeygi & Zarghami 2007, Wang *et al.* 2009, Saraswat *et al.* 2017, Nowicka-Bauer *et al.* 2018, Li *et al.* 2019). Johnson *et al*. (2012) showed that 2–9% of the human population are affected by a homozygous SNP in the gene for choline dehydrogenase which makes these individuals more susceptible to choline deficiency and that their sperm are less progressively motile with reduced levels of ATP. Our work suggests that it would be important to examine whether low choline in humans leads to higher oxidative stress and lower motility in sperm and whether choline supplementation can help in these cases.

Contrary to our hypothesis, we found little difference in the exogenous metabolome and degree of glycolysis between sperm from normozoospermic and asthenozoospermic ejaculates. Frequently, we found that data normalised to vitality or motility from 40% sperm populations exhibit greater glycolysis than 80% populations. Counterintuitively, ^13^C-NMR showed the lowest lactate production for 80N sperm despite having the highest progressive motility, whereas lactate levels for 40A sperm, with the lowest progressive motility, were significantly higher. This suggests that whilst (40% and 80%) sperm from asthenozoospermic ejaculates were metabolically similar to normozoospermic ones, the sperm population from 80N were more efficient and had less requirement to generate NADH in order to support further ATP production through glycolysis.

There is some ambiguity in the literature as to how lactate metabolism is affected by asthenozoospermia. Our study found no significant differences in lactate production between comparable asthenozoospermic and normozoospermic sperm populations; however, ‘40%’ sperm populations did produce more lactate than ‘80%’ sperm populations from labelled glucose, fructose and pyruvate when normalised to motile sperm. Ma*et al.* (2019) found that lactate was increased in blood plasma of men who produced asthenozoospermic ejaculates. In contrast, Zhao*et al.* (2018) found significantly reduced lactate levels in sperm from asthenozoospermic compared to normozoospermic ejaculates. However, their protocol (Percoll centrifugation, freezing followed by methanol extraction of pooled sperm samples normalised by total peak area) might account for differences in results. Therefore, further work is needed in understanding the importance of glycolysis in supporting the motility of asthenozoospermic samples.

Several other studies have indicated other key pathways and metabolites that are altered in sperm by asthenozoospermia. Notably, several proteomic studies have indicated reduced capacity for oxidative phosphorylation in asthenozoospermic samples (Amaral *et al.* 2014, Asghari *et al.* 2017, Nowicka-Bauer *et al.* 2018). On the other hand, Hereng *et al.* (2011) performed experiments on sperm from normozoospermic donors, where they showed that pyruvate (0–1.0 mM) was entirely converted to lactate and correlated with increased levels of ATP per million sperm. They also noted that under capacitating conditions, pyruvate did not enter the Krebs cycle. For our experimental conditions, a ^13^C-bicarbonate peak (formed by the enzymatic cleavage of ^13^C_1_ from pyruvate) was only observed sporadically. Radioactive tracer experiments have shown the production of ^14^CO_2_ by human sperm (Murdoch & White 1968, Ford & Harrison 1981). ^13^C-NMR is far less sensitive than this, and the observation of a bicarbonate signal suggests a large increase in OxPhos activity in particular sperm experiments. Whether this relates to incubation conditions (e.g. oxygenation), sperm status (e.g. capacitation) or is phenotype relating to the sperm of some men require further investigation. It is possible that pyruvate usage represents a secondary metabolic system that can rapidly produce co-factors necessary for further ATP generation when required (e.g. hyperactivation).

In general, sperm concentration correlated with lactate integral for ^13^C_u_-glucose and ^13^C_u_-fructose but not ^13^C_1_ – pyruvate, even though the range of sperm concentrations incubated with ^13^C_1_-pyruvate were comparable to the other substrates. Using faster and more sensitive, hyperpolarised NMR techniques showed that lactate derived from ^13^C_1_-pyruvate did correlate with sperm concentration (Reynolds *et al.* 2017*b*). Our previous study showed that the rate of conversion for ^13^C-pyruvate was more rapid and reached a plateau earlier than ^13^C-glucose, ^13^C-fructose which continued to increase over tens of hours (Calvert *et al.* 2019). This could explain why lactate integrals from ^13^C_1_-pyruvate were smaller than those from ^13^C_u_-glucose or ^13^C_u_-fructose, leading to a greater error in the correlation plot with sperm concentration.

To our knowledge, no studies have been made on the metabolism of non-sperm cells present in semen. Multiple linear regression shown here suggested a possible leukocyte contribution to the ^13^C_1_-lactate integral, although this was not consistent. The problem arises that the non-sperm cell concentration is calculated as a proportion of the sperm concentration and so may represent a cross-correlation to sperm (three of our sample groups showed a significant correlation between these sperm and non-sperm concentrations, Supplementary Table LMR2); however, these did not necessarily correspond with the linear model identifying a significant non-sperm correlation. Furthermore, the model assumes that all the non-sperm cells are metabolically active and further research is required to establish whether this is the case. In preliminary experiments, we investigated the use of magnetic bead-tagged antibodies (Dynabeads MPC-1, Thermo Scientific) to remove non-sperm cells, particularly important for the ‘40%’ sperm populations. These magnetic beads could not be fully removed from the samples and their presence caused NMR peak broadening, particularly of the water signal, and reduced the ability to measure other metabolites. Whilst important for removing seminal plasma (Reynolds *et al.* 2017*a*), extra washing of the sperm lengthens the sperm preparation protocol and introduces additional centrifugation stress on the sperm.

In conclusion, an 80% sperm population from asthenozoospermic ejaculates (80A sperm) showed significantly reduced choline metabolite peaks, compared to 80% sperm populations from normozoospermic ejaculates (80N sperm). There were no significant differences in the metabolome of 40A and 40N sperm, which implies that poor-quality sperm from asthenozoospermic or normozoospermic ejaculates are indistinguishable. Despite clear differences in progressive and total motility, glycolytic activity, as observed through lactate integrals, was similar irrespective of whether they were from asthenozoospermic or normozoospermic ejaculates when comparing DGC-separated populations (i.e. 40A vs 40N or 80A vs 80N). The principal difference in glycolysis existed between the ‘40%’ and ‘80%’ populations of either ejaculate type. However, if regarded as a continuum of sperm quality, then the significant difference could represent an inefficiency in energy production for sperm from asthenozoospermic ejaculates. It is also possible that glycolysis provides the underlying energy requirement for sperm function and only provides some of the ATP required for motility. Our work suggests that choline metabolism and supplementation are interesting avenues to explore for improving motility in asthenozoospermic ejaculates.

## Declaration of interest

The authors declare that there is no conflict of interest that could be perceived as prejudicing the impartiality of the research reported.

## Funding

This work was funded by the MRC Grant No. MR/M010473/1.

## Data availability

The data underlying this article will be shared on reasonable request to the corresponding author.

## Author contribution statement

S J C and S R jointly carried out experimental protocols and data analysis. All authors designed the study and contributed to the writing of the manuscript. S J W acted as a consultant for statistical analysis. A A P, S R and M N P proposed the study. A A P and M N P obtained the MRC grant funding.
